# Exercise and Oxidative Damage in Nucleoid DNA Quantified Using Single Cell Gel Electrophoresis: Present and Future Application

**DOI:** 10.3389/fphys.2016.00249

**Published:** 2016-06-22

**Authors:** Gareth W. Davison

**Affiliations:** Sport and Exercise Science Research Institute, Ulster UniversityBelfast, UK

**Keywords:** DNA strand breaks, DNA repair, comet assay, single cell gel electrophoresis, exercise-induced oxidative stress, free radical damage, antioxidant

## Abstract

High intensity exercise can enhance the production of reactive oxygen and nitrogen free radical species, which may cause a number of perturbations to cellular integrity, including deoxyribonucleic acid (DNA) modification. In the absence of adequate DNA repair, it is theoretically possible that several biological disorders may ensue, in addition to premature aging. This striking hypothesis and supposition can only be realized in the presence of sound methodology for the quantification of DNA damage and repair. The alkaline single-cell gel electrophoresis or “comet assay” is a simple and reliable method for measuring the components of DNA stability in eukaryotic cells. The assay is commonly used in research associated with genotoxicology and in human bio-monitoring studies concerned with gene-environment interactions; but is currently less appreciated and under-utilized in the domain of exercise science. No exercise related study for example, has incorporated the comet assay combined with fluorescent *in situ* hybridization methodology to detect and investigate whole genome, telomeric DNA, or gene region-specific DNA damage and repair in cells. Our laboratory and others have used the comet assay in conjunction with lesion-specific endonucleases to measure DNA strand breaks and oxidized bases to confirm that high intensity exercise can damage and destabilize DNA. Thus, the primary function of this review is to highlight recent advances and innovation with the comet assay, in order to enhance our future understanding of the complex interrelationship between exercise and DNA modification in eukaryotic cells. A brief synopsis of the current literature addressing DNA stability as a function of continuous aerobic exercise is also included.

## Introduction

### Exercise, free radical production and DNA damage

It is well accepted that exercise training is associated with a plethora of health benefits, such as a decrease in susceptibility to cancer, diabetes and cardiovascular disease (Warburton et al., 2006). Although regular exercise at a moderate intensity can activate important cell adaptive properties (Ristow et al., 2009), sporadic and strenuous bouts of exercise may induce oxidative stress due to an augmented production of reactive metabolites of oxygen (ROS) and nitrogen free radical species (RNS) (Packer et al., 2008). Exercise-induced free radical formation may impair cell function by oxidatively modifying nucleic acids, where DNA damage and insufficient repair may lead to genomic instability and a state of mutagenesis.

While the various mechanistic sources of free radicals attributable to DNA damage following exercise are not well understood, it is certain that the hydroxyl radical (^•^OH) plays an integral role. ^•^OH radicals are typically produced in cells by Fenton reactions that involve the reduction of H_2_O_2_ by either ferrous (*k* ~ 76 M^−1^ s^−1^) or copper ions (*k* ~ 4.7 × 10^3^ M^−1^ s^−1^) (Cadet et al., 2012; Halliwell and Gutteridge, 2015). ^•^OH-mediated DNA damage is initiated by electron or hydrogen abstraction or by ^•^OH reacting with a DNA base and the ribose sugar backbone at diffusion controlled rates (e.g., 2-deoxyguanosine: 5 × 10^9^ M^−1^ s^−1^; Chatgilialoglu et al., 2011). It is estimated that 70% of ^•^OH reacts with DNA bases, and 30% with deoxyribose moieties (Nikitaki et al., 2015). As Cobley et al. (2015) points out, the chemistry of ^•^OH-mediated DNA is inherently complex, and may be explained using a propagation type approach. It is currently understood that when ^•^OH attaches to a DNA base, DNA-centered radicals are produced. These radicals can subsequently react with oxygen (O_2_) or other free radical species such as superoxide (${\text{O}}_{2}^{•-}$) or nitric oxide (NO), to form a DNA oxidized end product (Ramirez et al., 2007). Accordingly, with the addition of ^•^OH onto a DNA base aromatic ring, there can be as many as 70 different oxidation end-products produced (Nikitaki et al., 2015).

**Figure 1:**
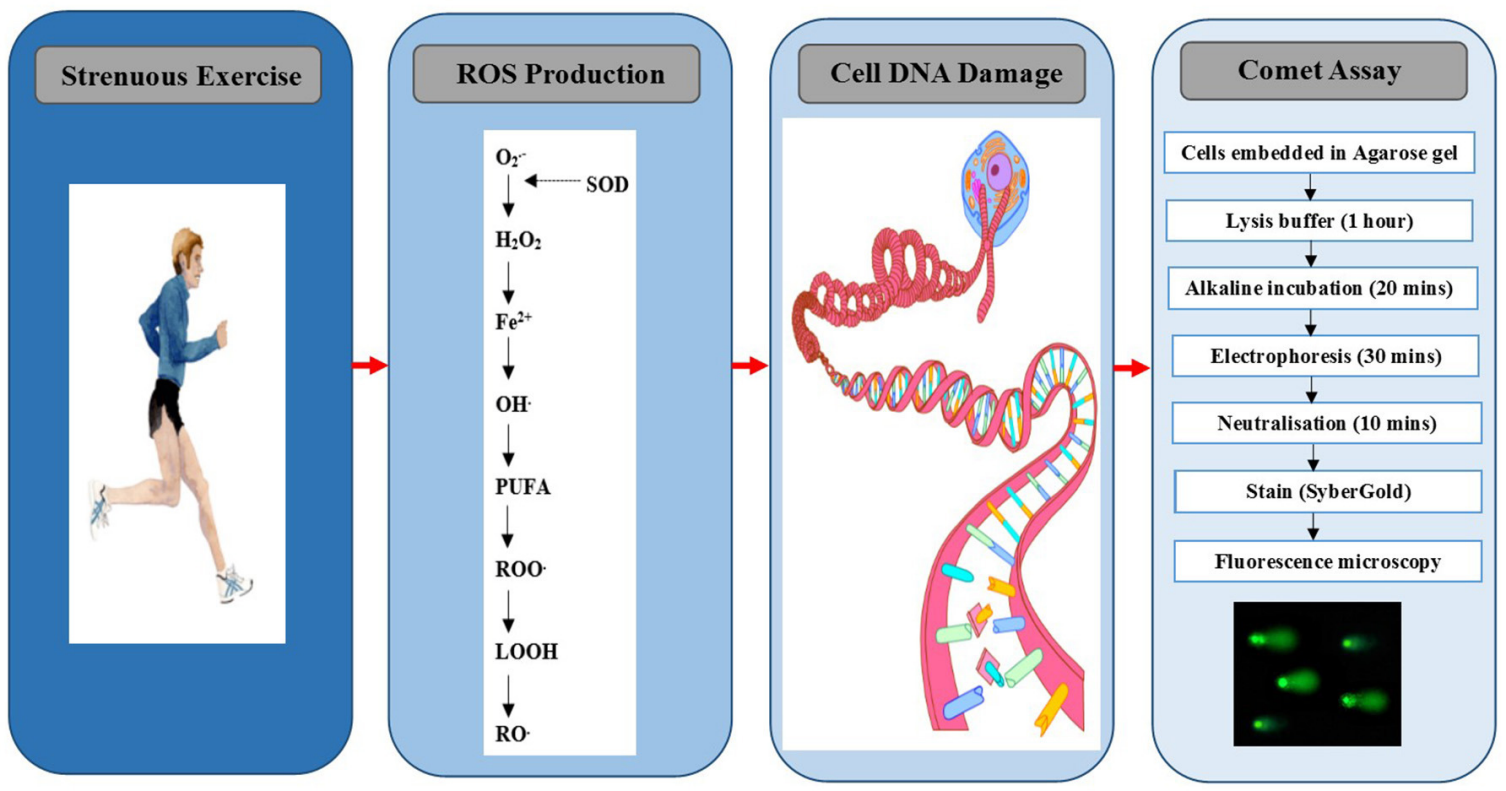
**Exercise-induced nucleoid DNA damage quantified by the comet assay**. ${\text{O}}_{2}^{-}$, superoxide; SOD, superoxide dismutase; H_2_O_2_, hydrogen peroxide, Fe^2+^, iron; OH, hydroxyl; PUFA, polyunsaturated fatty acid; ROO, peroxyl; LOOH, lipid hydroperoxide; RO, alkoxyl.

A number of assays have been developed over the years to quantify DNA free radicals directly (electron paramagnetic resonance spectroscopy and/or immuno-spin trapping), as well as the by-products of DNA damage and oxidation. With regard to the latter, one particular method that has been utilized, but not extensively, to quantify DNA damage following exercise is the single cell gel electrophoresis assay, otherwise known as the comet assay (Figure 1). The purpose of this review and indeed its novel feature, is to ascertain the use of the comet assay in human exercise studies, and to highlight recent advances associated with the assay. The final section will determine how this innovation can enhance our future understanding of the complex interrelationship between exercise and DNA modification.

### Comet assay background

The comet assay (alkaline single-cell gel electrophoresis) is regarded as a simple and sensitive method for measuring single and double-stranded DNA breaks in peripheral mononuclear cells (Azqueta and Collins, 2013). The concept of using electrophoresis as a method to determine DNA single strand breaks following relaxation of DNA supercoils was first devised by Ostling and Johanson in 1984 (Olive and Banáth, 2006). A few years later, a modified version using alkaline conditions was published by Singh et al. (1988), and as outlined by Olive and Banáth (2006), the assay became attractive to users due to (a) the fact that only 1000 cells were required, (b) the cells did not require tagging with a radio isotope, thus permitting the quantification of damage in any nucleated cell, and lastly (c) the method could prove useful in measuring variations in response to DNA damaging agents within the same or similar populations. In a later experiment using single cells, Olive et al. (1990) modified the original version of Ostling and Johanson's (1984) method, and demonstrated “comet like” images where the comet head contained high-molecular-weight DNA, whilst the comet tail showed the migrating fragments.

The principle of the comet assay involves embedding cells on agarose gel, which are lysed with detergent and a high concentrated salt solution in order to remove membranes, cytoplasm and most of the nuclear material aside from DNA. Even though histone proteins are eliminated, the winding of the DNA remains as a compact structure in the form of supercoiled loops attached to the nuclear matrix, and is referred to as a nucleoid. If supercoiling is relaxed by the presence of a single or double strand break, the loop of the DNA is free to migrate toward the anode when electrophoresis is applied, thus forming a comet tail. The appearance of this comet tail only forms when DNA loops have relaxed supercoiling by virtue of a break (Collins, 2015). Fluorescence microscopy is normally used to quantify the relative intensity of the comet tail which is directly proportional to the frequency of DNA strand breaks (Collins and Azqueta, 2012). Collins and Azqueta (2012) further postulate that the comet assay has the ability to resolve damage up to ~3 breaks per 10^9^ Da, while at a higher degree of damage, essentially all DNA loops are in a relaxed state and located within the tail.

## Comet assay application

### Lesion-specific endonucleases

Oxidation of bases in DNA can occur at a similar rate to DNA strand breaks (Collins et al., 2008). A modified version of the standard alkaline comet assay may be used to examine oxidized DNA bases, by detecting oxidized purines (guanine and adenine) and pyrimidines (thymine and cytosine) using lesion-specific enzymes. The modification involves an additional step to the standard assay, where following lysis of agarose-embedded cells, the nucleoid is digested with a lesion-specific endonuclease, such as formamidopyrimidine DNA glycosylase (FPG) for oxidized purine detection (principally 8-oxoguanine), or endonuclease III for evidence of pyrimidine oxidation. This process of enzyme digestion is designed to recognize a specific type of damage in the DNA and create a break (Collins, 2004). Base oxidation is ultimately determined by subtracting the score from a control incubation with buffer (DNA strand breaks) from the score with enzyme incubation, to give the score for “net enzyme-sensitive site damage” (Collins et al., 2008).

### Comet-FISH

The comet assay can be combined with fluorescent *in situ* hybridization (FISH) methodology to detect and investigate whole genome, telomeric DNA, centrometric DNA and gene region-specific DNA damage and repair in cells. Whereas the standard comet assay allows for the separating of fragmented from non-fragmented DNA, comet-FISH uses a hybridization step following electrophoresis, which permits the detection of labeled DNA sequences by using probes of cDNA or oligonucleotides (Collins, 2004; Glei et al., 2009). This technique allows for assignment of the probed sequences to the damaged (tail DNA) or undamaged (head DNA) part of the comet. When two fluorescence signals are detected with a probe for a particular gene in the head of a comet, this highlights that the gene is in the vicinity of intact and undamaged DNA in the nuclear matrix, whilst the appearance of a spot(s) in the tail of a comet suggests that DNA damage has occurred close to the site of the probed gene (Glei et al., 2009). It is therefore pertinent to highlight that the comet-FISH assay detects DNA damage and repair only within the vicinity of a probed gene, as oppose to quantifying actual gene modification. To date, the use of the comet-FISH assay has largely been confided to the study of DNA damage and repair within cells associated with cancer (McKelvey-Martin et al., 1998; McKenna et al., 2003), gene fragmentation resulting from x-ray irradiation (Amendola et al., 2006) and in studies interested in telomere behavior (Arutyunyan et al., 2004).

### Measuring DNA repair

Human cells contain a plethora of repair enzymes that have the ability to positively modify and correct DNA damage prior to it causing severe genomic instability. Indeed, cells have various DNA repair pathways, involving multiple enzymes that deal with a distinct type of damage. For example, where free radicals generated by metabolism are the prime culprits at initiating DNA damage, the base excision repair (BER) pathway is primarily associated with small base alterations (Azqueta et al., 2014).

The cellular repair assay or “challenge” assay, is regarded as one of the most simplistic and functional methods for quantifying DNA repair (Au et al., 2010), and it works on the premise of treating cells with a specific DNA-damaging agent such as H_2_O_2_, and subsequently monitoring the removal of the residual damage over time (Collins and Azqueta, 2014). Whilst the primary purpose of this assay is to monitor strand break rejoining, excision repair of oxidized bases can also be assessed by incorporating the digestion of DNA (nucleoids) with a lesion-specific enzyme such as formamidopyrimidine DNA glycosylase (FPG). This approach can ascertain the removal of DNA lesions, and more specifically the conversion of oxidized purines into strand breaks (Azqueta et al., 2011). As outlined by Shaposhnikov et al. (2011), it is possible to study the DNA repair of specific genes or DNA sequences by modifying the challenge assay to incorporate the basic tenets of the comet assay with fluorescent *in situ* hybridization (FISH).

An alternative assay at measuring DNA repair, is the comet-based *in vitro* repair assay by Collins et al. (2001), and this approach is favored for monitoring the response of lymphocytes to low levels of DNA damage. This particular assay works in converse to the cellular repair assay, where DNA nucleoids containing a specific lesion are incubated with a cell extract containing a certain amount of repair enzyme (Collins et al., 2001).

### Recent comet assay innovation and modifications

Since the original comet assay, devised by Ostling and Johanson (1984), there have been a multitude of developments and modifications, all designed to make the assay more versatile, innovative and user-friendly. Whilst not comprehensive in approach, a brief overview of the modifications closely aligned to blood collection, handling and processing are provided. Indeed, the following developments can be beneficial to exercise physiologists conducting research in a remote environment and when multiple sample analysis is required.

### Freezing whole blood

Preservation of cells for DNA damage quantification using the comet assay, conventionally involves the isolation of lymphocytes by centrifugation, suspension in freezing medium and slow freezing to −80°C. This methodology can be time consuming and laborious. Al-Salmani et al. (2011) showed that small volumes (~250 μl) of whole blood can be successfully stored at −80°C for up to 1 month without the use of a cryopreservative, and with no artifactual formation of DNA damage. This approach can also extend to the enzyme-modified comet assay. In a recent follow up study, Akor-Dewu et al. (2014) observed higher basal DNA damage in isolated leucocytes compared with whole blood stored for up to 11 months, however, FPG-sensitive sites were detected more efficiently. This modified method of sample handling can be most applicable when sample number is excessive, and volume is limited, or when blood samples are collected at sites remote from the laboratory (Akor-Dewu et al., 2014).

### High throughput sample processing

A plethora of new initiatives associated with rapid comet assay analysis have been developed over the last number of years, and these include:

1. *More samples on a glass surface*; According to Brunborg et al. (2014) the first comet assay commercial kit was available in 1999, where gel samples were separated by hydrophobic spacers. Since then, technology has allowed for a larger glass slide holding 96 samples; however, these large slides can add considerable costs to the assay. As a replacement to the traditional glass slide, a GelBond® polyester film can be used to support agarose gels in a 96-well format (Gutzkow et al., 2013). This innovation is relatively inexpensive and an effective method of processing multiple samples.
2. *Comet scoring*; Semi-automated scoring of comets can be extremely time consuming, particularly when it involves 96-spot scoring with up to 50 comets per sample (Brunborg et al., 2014). There are at least two automated comet scoring systems available (Imstar Pathfinder™ and MetaSystems CometImager), which require minimal operator interaction, and are superior in speed and tend to avoid operator-dependent bias compared to the semi-automated approach.

## Exercise and comet assay application

In a seminal investigation, Hartmann et al. (1994) demonstrated that exhaustive exercise on a treadmill was sufficient to induce DNA strand breaks 24 h post-exercise. Consistent with this, others have confirmed that either exhaustive running (Niess et al., 1996; Davison et al., 2005; Fogarty et al., 2013a) or cycling (Mars et al., 1998; Zhang et al., 2004) at or near *V*O_2max_, or indeed rowing (Sardas et al., 2012) can damage DNA strands immediately or 24 h following exercise; even though in a few cases trained subjects were used. Using a slightly reduced exercise intensity, Fogarty et al. (2011) demonstrate that cycling at 70% of oxygen capacity can cause DNA strand breaks, and in a follow up study, continuous maximal leg muscle contractions is also shown to induce DNA damage (Fogarty et al., 2013b). Using a similar exercise model, Gray et al. (2014) observed an increase in DNA damage following repetitive eccentric knee contractions, however, H_2_O_2_ stimulated DNA damage was lower immediately following exercise when fish oils were ingested. In an interesting study at altitude, Møller et al. (2001) observed an increase in DNA strand breaks at rest (compared with sea level) and following acute exercise (compared with pre-exercise). When the length of time or distance associated with exercise is increased, the majority of studies show enhanced DNA strand break damage. For example, single exercise bouts performed over 21.1 km (Niess et al., 1998), 42 km (Tsai et al., 2001), and 50 km (Mastaloudis et al., 2004), or over a fixed amount of time (2.5 h, Peters et al., 2006) all present an adverse effect on DNA stability either during or following exercise, irrespective of the mode. Consecutive bouts of endurance type exercise on DNA stand breaks have been investigated with mixed outcomes. Using a competitive short triathlon event, Hartmann et al. (1998) observed an increase in DNA migration up to 5 days post-exercise, while Wagner et al. (2010) showed a decrease in DNA migration following ultraendurance exercise. Contrary to this, Briviba et al. (2005) observed no change in DNA strand breaks following two consecutive bouts of endurance exercise (Table 1). Palazzetti et al. (2003) examined exercise training on DNA stability and demonstrated that DNA strand breaks can increase following overload training. It thus appears that acute high intensity, and more prolonged endurance bouts of exercise or exercise training can damage DNA, and this seems to be consistent across trained and untrained individuals or indeed if the exercise occurs in a state of either normoxia or hypoxia.

**Table 1:** **Literature highlighting exercise and DNA damage using the comet assay**.

**References**	**Exercise protocol**	**Blood type**	**Parameter**	**Effect**
Hartmann et al., 1994	Run to exhaustion	Leukocytes	SB's	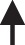 24 h post-run
Niess et al., 1996	Run to exhaustion	Leukocytes	SB's	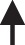 24 h post-run
Niess et al., 1998	21.1 km run	Leukocytes	SB's	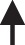 24 h post-run
Hartmann et al., 1998	1.5 km swim, 40 km cycle, 10 km run	Leukocytes	SB's, FPG-s s	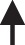 SB's 24 h until 5 days following exercise; 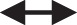 FPG-s s
Mars et al., 1998	Run to exhaustion	Lymphocytes	SB's	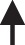 24 h post-run
Møller et al., 2001	Exhaustive bike test in normoxia and hypoxia	Lymphocytes	SB's, FPG-s s, ENDO III-s s	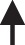 SB's immediately following exercise in hypoxia; 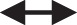 FPG-s s and ENDO III-s s after exercise in normoxia and hypoxia
Tsai et al., 2001	42 km run	PBMC	SB's, FPG-s s, ENDO-III-s s	SB's 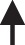 24 h post-run, FPG, and ENDO-III-s s 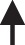 immediately post-run
Palazzetti et al., 2003	4 weeks of overload exercise	Leukocytes	SB's	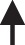 Immediately after overload exercise
Zhang et al., 2004	Exhaustion bike test	Leukocytes	SB's	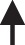 6 h and 24 h post-exercise
Mastaloudis et al., 2004	50 km ultra-run	Leukocytes	SB's	SB's 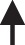 at half distance
Briviba et al., 2005	21.1 km and 41.2 km runs	Lymphocytes	SB's, FPG-s s, ENDO III-s s	ENDO-III-s s 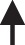 following both runs, SB's and FPG 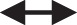 immediately after both runs
Davison et al., 2005	Run to exhaustion	PBMC	SB's	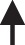 Following exercise
Peters et al., 2006	2.5 h run at 75% *V*O2max	Lymphocytes	SB's	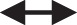 Immediately after and 3 h post-run
Tanimura et al., 2008	1 h cycling at 75% *V*O2max	Lymphocytes	SB's with hOGG1	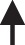 3 h post-exercise
Reichhold et al., 2009	3.8 km swim, 180 km cycle, 42 km run	Lymphocytes	SB's	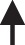 24 h post-exercise
Tanimura et al., 2010	3 × 1 h cycling at 75% *V*O2max	Lymphocytes	SB's with hOGG1	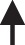 Over consecutive sessions
Wagner et al., 2010	3.8 km swim, 180 km cycle, 42 km run	Lymphocytes	SB's, FPG-s s, ENDO III-s s	SB's 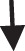 immediately post-exercise. ENDO III-s s 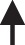 5 day post-exercise.
Fogarty et al., 2011	Bike test at 40, 70, and 100% *V*O2max	Leukocytes	SB's	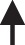 At 70 and 100% *V*O2max
Sardas et al., 2012	2000 M rowing at 80% peak power	Lymphocytes	SB's	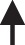 24 h post-exercise
Fogarty et al., 2013a	Run to exhaustion	Lymphocytes	SB's	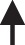 Following exercise
Fogarty et al., 2013b	100 isolated and maximal knee extension contractions	Lymphocytes	SB's	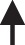 Following muscle contractions
Gray et al., 2014	200 eccentric knee contractions with and without fish oil ingestion	Lymphocytes	SB's, H_2_O_2_ stimulated damage	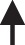 Immediately following muscle contractions (pooled group data); 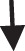 following exercise with fish oil ingestion

Parameters are indicated as strand breaks (SB's), formamidopyrimidine glycosylase-sensitive sites (FPG-s s), endonuclease III-sensitive sites (ENDO III-s s), human 8-oxoguanine DNA glycosylase (hOGG1). Effects indicated as 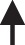 increase; 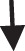 decrease; 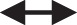 no change. Peripheral blood mononuclear cells (PBMC); Maximal oxygen uptake (VO2_max_).

Relatively few of the above studies have examined DNA lesions in the form of oxidized purines and pyrimidines. Using the comet assay, Hartmann et al. (1998) and Briviba et al. (2005) observed no change in FPG-sensitive sites; however, the latter study did detect a rise in ENDO III-sensitive sites following endurance exercise. This finding is supported by Wagner et al. (2010), and further by Tsai et al. (2001) who documented a change in both FPG and ENDO III-sensitive sites following continuous exercise. With regard to short duration exercise, Tanimura et al quantified DNA damage using the comet assay combined with hOGG1 following a single (2008) and consecutive (2010) bouts of high intensity exercise, and on both occasions DNA base damage increased. Even though DNA strand breakage occurs in hypoxia, there is no evidence that this translates into DNA base oxidation, as FPG and ENDO III do not change as a function of exercise (Møller et al., 2001).

## Conclusions and future application of the comet assay in exercise biochemistry

It is clear from a review of the available literature that high-intensity exercise can damage DNA, which is quantified in human cells using the comet assay. However, what is not so clear is whether exercise-induced DNA damage is physiologically important in that it may be counterproductive to human health, particularly when one considers that excessive damage to DNA is associated with human pathology. Whilst not related to exercise *per se*, Halliwell and Gutteridge (2015) eloquently articulates the consequences of damage to DNA by reactive species. DNA damage can inhibit DNA replication and cell division, and an excessive amount of damage can cause cell death via p53-mediated apoptosis and/or NAD^+^ depletion. The p53 gene in particular is an important tumor suppressor gene and a transcription factor protein that can detect DNA instability, and when it does, it puts the break on the cell cycle process and initiates DNA repair. Although the p53 gene is often referred to as “*the guardian of the genome*,” it is also the most frequently mutated gene in human cancers (Halliwell and Gutteridge, 2015). Whilst high-intensity exercise has the potential to cause DNA damage, we know little regarding the effects of p53 stability and its potential to be adversely disturbed following exercise. The comet assay along with fluorescent *in situ* hybridization can detect and investigate gene region-specific DNA damage in cells. Although the assay does not determine actual gene modification, but alternatively the DNA damage and repair within the vicinity of a probed gene, the assay can act as a first step to enhance our understanding of the interrelationship between exercise, DNA damage and possible gene disruption.

Complete repair of DNA damage is required for cell survival without excessive mutation (Halliwell and Gutteridge, 2015). Base excision repair removes damage within a base, by using DNA glycosylase enzymes to hydrolyse the bond linking the damaged base to the sugar-phosphate backbone. An example of a glycosylase enzyme in humans is OGG1 which removes 8OHG, leaving a apurinic or apyrimidinic mutagenic site in DNA. It is generally accepted that one of the best ways to characterize DNA stability *in vivo*, is to contextualize “steady-state” damage, which is essentially the balance between damage and repair. Hence, a rise in cellular DNA damage could be due to increased damage, decreased repair, or both (Halliwell and Gutteridge, 2015). In stating this salient point, the majority of studies concerning exercise and DNA stability, have not taken the approach of measuring DNA damage and repair simultaneously. The need to incorporate this step-change into future exercise and DNA stability studies is further accentuated by the necessity to accurately interpret data outcomes; which is augmented by using the same assay approach. To this authors knowledge, no study has exploited the comet assay to address the notion of exercise and DNA (in)stability from a steady state perspective (i.e., measuring both DNA and repair in tandem). The aforementioned approach may be achieved by using the basic comet assay (strand breaks) alongside a requisite modification to incorporate the *in vitro* repair assay.

## Concluding perspectives

The comet assay is a simple and reliable method for quantifying DNA stability in eukaryotic cells, but is under-utilized and less appreciated in studies relating to exercise science. In fact, the assay has only been utilized in blood lymphocyte cells, whilst there is a need to examine different cell types such as muscle, in order to fully appreciate the effects of exercise on DNA modification and redox biology. The domain of exercise-induced DNA damage is inherently complex, and it is clear that considerable work has yet to be accomplished for a full understanding of the specific consequences (if any) of DNA damage. Moreover, as ROS are important signaling molecules, it is conceivable that DNA damage (stress) may also be beneficial for an efficient adaptive cell response. As such, the dialog associated with this review should stimulate further investigation, and future work ought to use the comet assay to combine DNA damage parameters (strand breaks and nucleotide base modification) alongside a DNA repair approach, while much specificity may also be acquired by examining damage in close proximity to a particular gene of interest (Comet-FISH).

## Author contributions

The author confirms being the sole contributor of this work and approved it for publication.

### Conflict of interest statement

The author declares that the research was conducted in the absence of any commercial or financial relationships that could be construed as a potential conflict of interest. The reviewer BK and handling Editor declared their shared affiliation, and the handling Editor states that the process nevertheless met the standards of a fair and objective review.
